# An RGD-Conjugated Prodrug Nanoparticle with Blood–Brain–Barrier Penetrability for Neuroprotection Against Cerebral Ischemia–Reperfusion Injury

**DOI:** 10.3390/antiox13111339

**Published:** 2024-11-01

**Authors:** Ayijiang Taledaohan, Maer Maer Tuohan, Renbo Jia, Kai Wang, Liujia Chan, Yijiang Jia, Feng Wang, Yuji Wang

**Affiliations:** 1 Department of Medicinal Chemistry, College of Pharmaceutical Sciences of Capital Medical University, Beijing 100069, China; ayijiang227@ccmu.edu.cn (A.T.); marmar8187@163.com (M.M.T.); jiarenbo0313@163.com (R.J.); wangkaiccmu@163.com (K.W.); chanliujia@mail.ccmu.edu.cn (L.C.); 18801239537@163.com (Y.J.); 2 Department of Medicinal Chemistry, Beijing Area Major Laboratory of Peptide and Small Molecular Drugs, Beijing Laboratory of Biomedical Materials, Engineering Research Center of Endogenous Prophylactic of Ministry of Education of China, 10 Xi Tou Tiao, You An Men, Beijing 100069, China

**Keywords:** cerebral ischemia–reperfusion injury, neuroprotection, LA-1, oxidative stress, proteomic analysis, integrin αvβ3, PI3K/Akt pathway, reactive oxygen species (ROS)

## Abstract

Cerebral ischemia–reperfusion injury significantly contributes to global morbidity and mortality. Loganin is a natural product with various neuroprotective effects; however, it lacks targeted specificity for particular cells or receptors, which may result in reduced therapeutic efficacy and an increased risk of side effects. To address the limitations of loganin, we developed LA-1, a novel compound incorporating an Arg-Gly-Asp (RGD) peptide to target integrin receptor αvβ3, enhancing brain-targeting efficacy. LA-1 exhibited optimal nanoscale properties, significantly improved cell viability, reduced ROS production, and enhanced survival rates in vitro. In vivo, LA-1 decreased infarct sizes, improved neurological function, and reduced oxidative stress and neuroinflammation. Proteomic analysis showed LA-1 modulates PI3K/Akt and Nrf2/HO-1 pathways, providing targeted neuroprotection. These findings suggest LA-1’s potential for clinical applications in treating cerebral ischemia–reperfusion injury.

## 1. Introduction

In 2019, stroke was responsible for a high mortality rate of 11.6% and a disability rate of 5.7%, making it the second-leading cause of death and the third-leading cause of disability globally. Ischemic strokes account for 62.4% of all stroke cases [1]. Currently, the primary treatments for acute ischemic stroke are intravenous rtPA injection and thrombectomy [2,3], which aim to reperfuse the occluded area and salvage the ischemic penumbra [4]. However, rtPA is associated with a risk of intracerebral hemorrhage and must be administered within a 4.5 h window [5], with delays reducing its efficacy [6]. Despite the potential for acute or chronic brain ischemia to induce neuroprotection and prevent long-term neuronal damage [7], all clinically validated neuroprotective drugs have failed to date [8]. This underscores the urgent need to develop effective treatments for brain ischemia.

Recent studies have demonstrated that compounds extracted from natural plants effectively treat various neurological disorders [9]. Loganin, a natural compound sourced from traditional Chinese medicinal plants such as Loasaceae, Gentianaceae, Rubiaceae, Cornaceae, and Caprifoliaceae [10,11,12,13,14], exhibits potent neuroprotective effects in cardiovascular and cerebrovascular diseases, including Alzheimer’s disease, Parkinson’s disease, and ischemia–reperfusion injuries [15,16,17,18]. Seung-Hwan Kwon et al. demonstrated that pre-treatment with loganin significantly improves cell viability, reduces LDH release and ROS production, and increases intracellular mitochondrial membrane potential (MMP) in SH-SY5Y cells subjected to H_2_O_2_-induced oxidative stress. Mechanism studies displayed that loganin impacts the phosphorylation of JNK/p38 and ERK 1/2 and modulates the Bcl-2/Bax apoptosis signaling pathway [19], indicating its potential as a neuroprotective agent.

The blood–brain–barrier (BBB) presents a significant challenge for drug delivery to the brain. To overcome this obstacle, specific brain-targeting ligands can bind to endogenous receptors within the brain, facilitating the transport of drugs across the BBB [20]. Integrin αvβ3 is a transmembrane glycoprotein composed of α and β subunits, which connect intracellular cytoskeletal elements to extracellular matrix molecules [21]. This integrin is believed to play a role in maintaining vascular integrity and remodeling during brain ischemia. It was demonstrated that an increased level of αvβ3 appears in the ischemic brain region at 2 h after middle cerebral artery occlusion (MCAO) [22]. Integrin αvβ3 binds to the Arg-Gly-Asp (RGD) sequence of its natural ligands, such as vitronectin, fibrinogen, osteopontin, and bone sialoprotein [23]. Accordingly, RGD-containing peptide analogs or recombinant proteins have been designed to target αvβ3 [24,25]. Tian et al. designed RGD-containing extracellular vesicles (EV) by attaching an RGD-containing recombinant protein to the EV surface. The RGD-EV exhibited the potential to target ischemic brain lesions and suppress post-stroke inflammation [26]. Further study demonstrated that the brain targeting delivery of RGD-EV was due to the binding of RGD-containing peptide to integrin αvβ3 [27].

Based on the above information, we designed an integrin αvβ3 mediated ischemic brain regions delivery agent LA-Arg-Gly-Asp-Val (LA-1) via conjugating RGD-containing peptide and loganin. This design is aimed to enhance brain accumulation and improve the neuroprotective efficacy of LA-1.

## 2. Materials and Methods

### 2.1. Materials

Loganin (CAS: 18524-94-2) and loganic acid (CAS: 22255-40-9) were obtained from Chengdu Push Bio-technology (Chengdu, China). c(RGDfk) (CAS: 161552-03-0) was obtained from MedChemExpress. Edaravone (CAS: 89-25-8) was obtained from Sigma Aldrich (Shanghai, China) Trading Co. (Shanghai, China). 5-Diphenyltetrazolium bromide (MTT, CAS: 298-93-1) was obtained from Sigma Aldrich (China). Fetal bovine serum (FBS, R35-076-CV) was purchased from Corning Co., Ltd. (New York, NY, USA). PBS buffer (KGL2206-500) and DMEM medium (KGL1206-500) were purchased from KeyGEN Biotech Co., Ltd. (Nanjing, China). Trypsin-EDTA (0.25%, BL512A) was purchased from Biosharp Life Sciences Co., Ltd. (Anhui, China). All reagents for proteomics were purchased from Thermo Fisher Scientific Co., Ltd. (Shanghai, China).

### 2.2. Synthesis of LA-1

The Arg(NO_2_)-Gly-Asp(OBzl)-Val-OBzl was prepared according to the literature procedures [28]. Under general environmental conditions, LA-Arg-Gly-Asp-Val (LA-1) was prepared by reacting loganic acid with Arg (NO_2_)-Gly-Asp(OBzl)-Val-OBzl to afford the LA-1 with protecting groups. The target compound of LA-1 was obtained under hydrogenolysis conditions. This followed the coupling reaction (details in Supporting Information Scheme S1). The final yield of the LA-1 was 14.2% and its purity exceeded 98% as determined by LC–MS.

### 2.3. Characterization of LA-1 and Loganin

#### 2.3.1. Fourier Transform Infrared Spectrometer (FT-IR)

The FT-IR spectra of LA-1 and loganin were recorded using a Nicolet IS5 instrument (Thermo Scientific, Waltham, MA, USA).

#### 2.3.2. UV–Visible Spectroscopy

In total, 1 mg of LA-1 or loganin was separately dissolved in 1 mL of deionized water and diluted to a concentration of 0.025 mg/mL. Then, all spectra of the compounds were recorded using a UV spectrophotometer (UV-2600, Shimadzu, Kyoto, Japan).

#### 2.3.3. NMR Spectrometer

The LA-1 was freeze-dried, and 5 mg was dissolved in 500 µL of DMSO-d6. Proton (1H) spectra were collected using an AVANCE 300 spectrometer (Avance II 300 MHz NMR, Bruker, Billerica, Germany).

#### 2.3.4. Particle Size and Zeta Potential

The size distribution and zeta potential of LA-1 and loganin were determined using a laser nanoparticle sizer (Nano-ZS90, Malvern Instruments Ltd., Malvern, Worcestershire, UK).

#### 2.3.5. Morphology

Each sample was further diluted to 0.1 mg/mL, and 10 μL was placed dropwise onto copper grids and dried at room temperature for 7 days. Transmission electron microscopy (TEM, Hitachi, JEM-2100, Tokyo, Japan) and scanning electron microscopy (SEM, Hitachi, S-4800, Tokyo, Japan) were used to observe the morphology. In total, 1 mg of LA-1 or loganin was ultrasonically dispersed in 1 mL of deionized water. Then, 100 μL of the samples were dropped onto 10 × 10 mm mica sheets and air-dried overnight. The surface morphology was characterized by atomic force microscopy (AFM, Bruker, Multimode 8, Bruker, Billerica, Germany).

### 2.4. Molecular Docking

To prepare the small molecule ligand, Discovery Studio (DS) software 2017 R2 should be opened first, and a new molecular window should be created. The structure of LA-1 should be imported into DS. Then, the “Prepare Ligands” and “Minimize Ligands” options should be used to assign charges and force fields to the ligand molecules.

Open the αvβ3 protein crystal structure (1L5G.pdb) downloaded from the Protein Data Bank (PDB) for the receptor protein. Select and delete the water molecules present in the protein structure. From the “Macromolecules|Prepare Protein” menu, select “Clean Protein” and ensure all 1L5G molecules are included. Next, navigate to “Receptor-Ligand Interactions|Define and Edit the Binding Site”, click “Define Receptor”, and set it as the receptor molecule in the docking system. Choose the original ligand molecule within the protein and use “From Current Selection” to define the sphere at the binding site.

Proceed to perform the CDOCKER calculations. Access “Ligand Receptor Interaction|Dock Ligands” and click “Dock Ligands (CDOCKER)” to open the parameter browser. In the browser, set the Input Receptor parameter by selecting 1L5G from the drop-down list to designate it as the receptor protein. For the Input Ligands parameter, choose “Molecule: All” from the drop-down list to specify the docking ligand. Adjust the Top Hits parameter to 10 and enter 0.5 in the Pose Cluster Radius parameter. Finally, click “Run” to execute the docking process.

### 2.5. In Vitro Activity

#### 2.5.1. Antioxidant Capacity Assay

The steps for the Electron Spin Resonance (ESR) assays were as follows: For the nitric oxide (NO) radical scavenging assay, 5 μL of 1.25 × 10^−2^ M ferrous sulfate (FeSO_4_·7H_2_O) solution, 5 μL of 2.5 × 10^−2^ M N-methyl-D-glucamine dithiocarbamate (MGD) solution, 5 μL of 1 × 10^−3^ M S-nitroso-N-acetylpenicillamine (SNAP) solution, and 5 μL of various drug solutions were sequentially added into a 0.5 mL centrifuge tube. For the hydroxyl radical (·OH) scavenging assay, 5 μL of 1.25 × 10^−2^ M ferrous sulfate (FeSO_4_·7H_2_O) solution, 5 μL of 2.5 × 10^−2^ M N-methyl-D-glucamine dithiocarbamate (MGD) solution, 5 μL of various drug solutions, and 5 μL of 1% hydrogen peroxide (H_2_O_2_) were sequentially added into a 0.5 mL centrifuge tube. For the 2,2-diphenyl-1-picrylhydrazyl (DPPH) free radical scavenging assay, 10 μL of 2 × 10^−3^ M DPPH solution was mixed with 10 μL of various drug solutions in a 0.5 mL centrifuge tube. Briefly, each mixture was transferred to a capillary tube after vortex mixing and then moved to a paramagnetic tube. All steps were completed within 2 min. The ESR spectra were collected using a spectrometer (FA-300, JEOL, Tokyo, Japan).

#### 2.5.2. Cell Viability Assay

Mouse brain microvascular endothelial cells (BEnd.3) were cultured in DMEM medium containing 10% FBS and maintained at 37 °C in a humidified atmosphere with 5% CO_2_. Drug solutions were prepared by diluting in PBS to the desired concentrations. Cells were seeded at a density of 4000 cells per well in 96-well plates. Once the cells had adhered, 25 μL of LA-1 or loganin solutions at varying concentrations were added to each well, followed by a 24 h incubation. BEnd.3 cells were treated with 3.12 or 6.25 μM of LA-1 or loganin for 24 h, followed by exposure to 200 μM tertiary-butylhydroperoxide (TBHP) for 4 h to induce oxidative stress. After the treatments, 25 μL of MTT solution (5 mg/mL) was added to each well, and the cells were incubated for an additional 4 h. The supernatant was then carefully removed, and the formazan crystals were dissolved in 150 μL of DMSO. Plates were shaken at room temperature for 15 min, and absorbance was measured at 490/570 nm using a microplate reader (Molecular Devices, SpectraMax ABS, San Jose, CA, USA).

Cell viability = (Absorbance (drug well))/(Absorbance (control well)) × 100%

#### 2.5.3. Evaluation of LA-1 Uptake in BEnd.3 Cells

BEnd.3 cells were seeded in 60mm dishes at 2 × 10⁶ cells/mL, with 5 mL of DMEM containing 10% FBS. After 12 h, the medium was replaced with 5 mL fresh medium, followed by 1 mL of 6.25 μM LA-1 solution. Cells were incubated for 3 or 6 h, washed twice with PBS, and harvested using 1 mL of HPLC-grade methanol. After vortexing for 2 min and centrifuging at 13,300 rpm for 5 min (6766-HS corning^®^ High Speed Microcentrifuge, Corning, NY, USA), the supernatant was concentrated and reconstituted with 200 μL methanol. Samples were centrifuged again at 13,300 rpm for 10 min, and the supernatant was analyzed via LC–MS (ACQUITY UPLC I-Class/Xevo TQ-XS, Waters, Milford, MA, USA). Standard curves were prepared by serial dilutions of HPLC-grade methanol with concentrations ranging from 1 μg/mL to 100 pg/mL for each LA-1.

#### 2.5.4. Assessment of the Impact of Integrin αvβ3 Inhibitor c(RGDfK) on LA-1 Uptake in BEnd.3 Cells

BEnd.3 cells were seeded at 2 × 10⁶ cells/mL in 6 cm^2^ dishes, with 5 mL DMEM containing 10% FBS. After 12 h, the medium was replaced with 5 mL fresh medium and 1 mL 20 nM c(RGDfK). After 1 h, 1 mL of 6.25 μM compound solution was added, followed by incubation for 3 or 6 h. Cells were washed twice with PBS, collected in 1 mL of HPLC-grade methanol, and centrifuged at 13,300 rpm for 5 min (6766-HS corning^®^ High Speed Microcentrifuge, Corning, NY, USA). The supernatant was concentrated and reconstituted in 200 μL methanol before a final 10 min centrifugation. The supernatant was then used for LC–MS/MS (ACQUITY UPLC I-Class/Xevo TQ-XS, Waters, Milford, MA, USA) analysis.

#### 2.5.5. ROS Depletion in TBHP-Induced BEnd.3 Cells

To monitor the depletion of intracellular ROS in the TBHP-induced BEnd.3 cell model, a 2′,7′-dichlorodihydrofluorescein diacetate (DCFH-DA) assay was employed. Initially, BEnd.3 cells were incubated with various therapeutic agents. Following this, oxidative stress was induced using 150 μM TBHP. The cells were then stained with DCFH-DA for 30 min and Hoechst for nuclear staining. Subsequently, the intracellular DCFH fluorescence intensity was analyzed using image flow cytometry (ImageStreamX MkII instrument, Amni, Luminex, Genk, Belgium) and flow cytometry (Becton, Dickinson and Company, SORP 4Laser-11Channel, Franklin Lakes, NJ, USA) to evaluate the levels of intracellular ROS. These methods comprehensively assessed the antioxidant effects of LA-1 and loganin against oxidative stress.

### 2.6. In Vivo Activity

#### 2.6.1. The Middle Cerebral Artery Ischemia (MCAO) Model

Male SD rats were purchased from Beijing Vital River Laboratory Animal Technology Co., Ltd. The animal studies were approved by the Institutional Animal Care and Use Committee of Capital Medical University, with the ethics number AEEI-2020-188. Humane care was provided to all animals, following the protocol and the Regulations on Laboratory Animal Welfare issued by the Chinese Ministry of Science and Technology.

A selection of 6–8-week-old Sprague–Dawley (SD) male rats weighing 250 to 280 g were divided into five groups, with 12 rats in each group: sham group, model group (MCAO), edaravone group (3 mg/kg), LA-1 group (10 mg/kg), and loganin group (30 mg/kg). In all groups of rats, anesthesia was induced with 3% isoflurane and then maintained at 2.5% isoflurane during surgery. Briefly, a 1–2 cm incision was made along the midline of the rat’s neck to expose the right carotid artery. Then, a nylon suture (A5-243650, Beijing Cinontech Co. Ltd., Beijing, China) coated with silica gel was inserted through the external carotid artery (ECA) into the middle carotid artery (MCA), which was then occluded. In this instance, the monofilament was not inserted in the sham group. After 2 h, the nylon suture was removed to allow reperfusion, and different drugs were immediately administered to the rats via the tail vein. Additionally, the sham and model groups received an equivalent dose of distilled water, with a dosage of 2 mL/kg. Cerebral blood flow was measured using a laser speckle flow imaging system (RWD Life science Co., Ltd., RFLSI ZW, Shenzhen, China) at three distinct time points: before ischemia, 2 h after ischemia, and 22 h after reperfusion. Neurological function scores for the treatment groups were assessed blindly at 22 h post-MCAO using the ZeLonga scoring system, after which the anesthetized rats were quickly euthanized. The brain tissue was harvested and then sectioned into seven slices, with six slices subjected to 2,3,5-triphenyltetrazolium chloride (TTC) staining to calculate the brain infarct area, while the remaining slice was preserved for future use.

#### 2.6.2. In Vivo Pharmacokinetic Studies

We purchased male Sprague-Dawley (SD) rats weighing between 200 and 220 g, which had been catheterized in the jugular vein, from Beijing Sinocon Pharmaceutical Technology Co., Ltd. (Beijing, China). The rats were divided into two groups: LA-1 group and loganin group, with three rats in each group. First, 200 μL of whole blood was collected from each rat at time point zero as a blank control. Subsequently, LA-1 and loganin were administered tail via injection at a dose of 30 mg/kg, dissolved in 0.9% saline. For the pharmacokinetics of the intravenously administered drugs, blood samples of 200 μL were collected from the rats at 5, 15, 30, 45 min, 1, 1.5, 2, 3, 6, 8, 12, and 24 h, using heparinized anticoagulation tubes for processing [29]. After each blood collection, 200 μL of perfusion solution was injected into the rats through the catheter to maintain their physiological status. To obtain the rat plasma, the samples were centrifuged immediately at 3500 rpm for 10 min (5810R) after collection. Methanol (300 μL) was added to plasma samples (100 μL) to precipitate plasma proteins, then rotary evaporation at 35 °C was used to remove methanol from the samples. The LA-1 and loganin were quantified by LC–MS/MS analysis (ACQUITY UPLC I-Class/Xevo TQ-XS, Waters, Milford, MA, USA).

#### 2.6.3. Intra-Brain Distribution Experiment

To investigate the distribution of LA-1 and loganin within the brain, the experimental protocol was as follows: First, the rats were subjected to 2 h of induced ischemia, after which the filament was removed to allow for reperfusion. Subsequently, LA-1 (10 mg/kg) and loganin (30 mg/kg) were administered via tail vein injection. Based on pharmacokinetic data, the concentrations of LA-1 and loganin in the blood were found to be negligible 3 h post-injection. At the 3 h time point, the rats were anesthetized, perfused with saline via the heart, and then euthanized to collect brain tissue. The brain tissue was divided into left and right hemispheres, with the right hemisphere representing the ischemic region. The right hemisphere was placed into a 5 mL centrifuge tube, and 600 μL of pre-chilled PBS was added, followed by homogenization on ice. A 200 μL aliquot of the homogenate was combined with 200 μL of chromatography-grade methanol and 200 μL of chromatography-grade acetonitrile, and subjected to ultrasonic treatment for 5 min. The mixture was then centrifuged at 13,000 rpm for 10 min at 4 °C, and the supernatant was collected for concentration. After concentration, 200 μL of chromatography-grade methanol was added for reconstitution, followed by another round of high-speed centrifugation to obtain the supernatant. Finally, the target molecules were quantified using LC–MS/MS (ACQUITY UPLC I-Class/Xevo TQ-XS, Waters, Milford, MA, USA). This series of steps allowed for a detailed assessment of the distribution of LA-1 and loganin in the rat brain.

#### 2.6.4. Magnetic Resonance Imaging (MRI)

In the animal experiment described in Section 2.6.1, following 2 h of ischemia and 22 h of reperfusion in the right cerebral tissue of rats, the model group, LA-1 group, and loganin group underwent MRI imaging. At the designated time points, the animals from each group were placed in a transparent anesthesia chamber and anesthetized using a mixture of 4% isoflurane and oxygen. The rats were monitored to ensure they remained calm. Once the animals were tranquilized, they were quickly removed and positioned on the scanning bed. During imaging, a constant-temperature heating system maintained the animals’ body temperature at 37 °C. A respiratory monitoring pad was placed under the abdomen to track respiratory parameters. Imaging commenced when the respiratory rate stabilized at 50 breaths per minute. The experiment utilized the Pharmascan 7T small animal magnetic resonance imaging system (Bruker, Billerica, Germany). The T2-weighted imaging parameters were set as follows: Field of View (FOV) = 3.30 cm × 3.30 cm, Matrix (MATRX) = 256 × 256, Repetition Time (TR) = 5600 ms, Echo Time (TE) = 36 ms, Flip Angle = 180°, Slice Thickness = 0.7 mm, Slice Distance = 0 mm, Number of Slices = 38, Acquisition Time (TA) = 8 min and 57 s, Number of Averages = 3. After imaging, the rats were returned to their cages to recover naturally.

#### 2.6.5. Hematoxylin-Eosin and Nissl Staining

Fresh brain, heart, liver, spleen, and kidney tissues from rats were fixed in 4% paraformaldehyde, dehydrated, embedded in paraffin, and sectioned into 4 µm slices. For HE staining, the sections were deparaffinized using a dewaxing agent (G1128, Solarbio, Beijing, China), rehydrated in ethanol of various concentrations, stained with hematoxylin and eosin, dehydrated, and sealed with neutral resin. For Nissl staining, thin sections were immersed in the staining solution for 15 min (G1086, Solarbio, Beijing, China), washed with water, rapidly dehydrated with absolute ethanol, and cleared in xylene for 5 min. The sections were then mounted with neutral resin. Finally, the sections were observed using a light microscope (Nikon Eclipse E100, Tokyo, Japan) and analyzed with a 3D viewer.

#### 2.6.6. TUNEL Staining

Paraffin sections were prepared as described for HE staining. The sections were dewaxed and antigen retrieval was performed using citric acid (pH 6.0). The sections were blocked in 0.1% Triton in PBS for 15 min. TUNEL reaction solution (TDT:dUTP:buffer = 1:5:50) was added, followed by blocking with BSA. After washing, sections were incubated with the secondary antibody (Cy3-goat anti-rabbit, GB21303, 1:300) at room temperature for 50 min. Finally, after PBS washing, the sections were stained with DAPI, mounted with an anti-fading mounting medium, and images were acquired using a fluorescence microscope (Nikon Eclipse C1, Tokyo, Japan).

#### 2.6.7. Enzyme-Linked Immunosorbent Assay (Elisa)

To quantify various cytokines and antioxidants in brain tissue, we employed the Enzyme-Linked Immunosorbent Assay (ELISA). Specifically, we measured levels of interleukin-1 beta (IL-1β, F2923-A, FANKEW, Shanghai, China), tumor necrosis factor-alpha (TNF-α, F3056-A, FANKEW, Shanghai, China), interleukin-6 (IL-6, F3066-A, FANKEW, Shanghai, China), superoxide dismutase (SOD, F3262-A, FANKEW, Shanghai, China), catalase (CAT, F8502-A, FANKEW, Shanghai, China), and glutathione (GSH, F3477-A, FANKEW, Shanghai, China). We followed the manufacturer’s instructions using commercially available ELISA kits to ensure accurate and reproducible results. First, the brain tissue was equilibrated at room temperature, followed by rinsing with saline, and the surrounding moisture was blotted dry with filter paper. A 50 mg sample of each tissue was taken, and 500 μL of PBS buffer (KGL2206-500) was added. The sample was homogenized on ice. The homogenate was centrifuged at 5000 rpm for 15 min, and the supernatant was collected for analysis. The absorbance was measured at a specific wavelength using a microplate reader to quantify the levels of IL-1β, TNF-α, IL-6, SOD, CAT, and GSH in the brain tissue samples.

#### 2.6.8. Sample Preparation for Proteomics

Fresh brain tissue samples were frozen at −20 °C for 20 min and then coronally sectioned into seven equal parts. The third slice was divided into left and right hemispheres, which were placed into separate centrifuge tubes. The right hemisphere (ischemic side) was homogenized in 200 μL of PBS on ice, with four rounds of homogenization, each lasting 15 s. A 50 μL aliquot of the homogenate was mixed with 200 μL of pre-chilled PBS buffer (KGL2206-500), vortexed for 60 s, and centrifuged using a microcentrifuge for 60 s. The supernatant was reserved for further analysis. A 2 μL sample of the supernatant was used to measure protein concentration with a BCA Protein Assay Kit (P0011, Beyotime Co. Ltd., Shanghai, China). Twenty microliters of homogenate were mixed with 60 μL of PBS, 20 μL of 2% SDS, 2 μL of TCEP, and 5 μL of CAA, and the mixture was heated to 99 °C for 10 min. Five volumes of cold acetone were added to the mixture to precipitate proteins, which were then incubated at −20 °C overnight. The next day, the mixture was centrifuged at 19,083 g for 10 min at 4 °C. The supernatant was collected and concentrated using a parallel evaporator. In the sample tube, proteins were digested overnight with 50 μL of tris-base buffer, 1 μL of calcium chloride, and 4 μL of trypsin enzyme at 37 °C. Digestion was verified by Coomassie brilliant blue staining. After digestion, the pH of the samples was adjusted to 2 with 2% TFA, and they were desalted using a C18 centrifugal column (89870, Thermo Scientific, Waltham, MA, USA). The samples were then quantified using the Peptide Quantitative Colorimetric Peptide Assay (23275, Thermo Scientific, Waltham, MA, USA). The samples were analyzed using a mass spectrometer (Orbitrap Exploris 480, Thermo Fisher, Waltham, MA, USA).

#### 2.6.9. Liquid Chromatography Tandem Mass Spectrometry Analysis

The LC–MS/MS detection system consisted of a nanoflow high-performance liquid chromatography (HPLC) instrument (Easy nLC1200, Thermo Fisher, Waltham, MA, USA) coupled to an Orbitrap Exploris 480 mass spectrometer (Thermo Fisher, USA). In the MS1 (primary mass spectrometry) scan, the resolution was set to 60,000, and the scan range was 350–1500 *m*/*z*. The AGC target (Normalized AGC Target) was set at 300, with a maximum injection time of 50 milliseconds. These parameters ensured sufficient ion accumulation to achieve optimal signal strength without overloading the detector. The microscan number was set to 1, with each scan cycle performing one microscan to expedite the acquisition speed.

In the MS2 (secondary mass spectrometry) scan, the scan range was divided into multiple windows, and the scans were scanned sequentially at a resolution of 15,000. Each isolation window had a width of 1.6 *m*/*z*, and the HCD (high-energy collision dissociation) collision energy was set to 30%. The AGC target was set at 75, with a maximum injection time of 22 milliseconds. The loading amount of each sample was kept at 1 μg. A self-packed column (150 μm inner diameter, ReproSil-Pur C18-AQ, 1.9 μm; (Dr. Maisch, Ammerbuch, Germany) with a length of 20 cm was used for peptide separation. The flow rate was maintained at 700 nL/min, the effective elution gradient of MS phase B (80% ACN) ranged from 1% to 99%, and the gradient duration was 120 min. Details of the gradient are shown in Supplementary Table S1.

The raw data files obtained from the mass spectrometer were imported into Thermo Proteome Discoverer 2.4.1.15 software for processing. The FDR threshold for protein and peptide levels was set at 0.01. Peak areas at the MS1 level were selected for protein and peptide quantification. Subsequent in-depth processing was performed using www.bioladder.cn, accessed on 12 May 2024.

### 2.7. Western Blot

Protein extraction from rat brain tissue samples was performed according to the instructions of the Protein Extraction Kit (GenePool/GPP1815). The protein concentration was adjusted using water and SDS-PAGE Loading Buffer (5×, GenePool/GPP1820) based on the required loading volume for electrophoresis. The samples were then boiled at 100 °C for 10 min to denature the proteins. Each sample was loaded with 35 μg of protein for SDS-PAGE gel electrophoresis. After electrophoresis, the proteins were transferred to a PVDF membrane via wet transfer. The membrane was then incubated with the primary antibody, diluted in 1% BSA or 5% milk. After primary antibody incubation, the membrane was washed three times with TBST (GenePool/GPP1822), each wash lasting 5 min. The secondary antibody was diluted in Milk Blocking Buffer (GenePool/GPP1819) and incubated with the membrane. Following incubation, the membrane was washed again with TBST four times, each for 5 min. Finally, the PVDF membrane was immersed in ECL solution (GenePool/GPP1824) for 1 min, followed by exposure, development, and fixation in a dark room. Primary antibodies used were: Bax (Abcam/32503, 1:1000), Bcl-2 (Abcam/194583, 1:1000), PI3K p85 alpha (Abcam/ab191606, 1:1000), PI3K p85 alpha (phospho Y607) (Abcam/ab182651, 1:1000), Akt (CST/9272, 1:1000), Phospho-Akt (CST/4060, 1:1000), Nrf2 (Proteintech/16396-1-AP, 1:1000), Heme Oxygenase 1 (HO-1) (Abcam/ab68477, 1:500), GAPDH (Abcam/ab181602, 1:3000), goat anti-mouse IgG-HRP (Abcam/ab6789, 1:5000), and goat anti-rabbit IgG-HRP (Abcam/ab6721, 1:5000).

### 2.8. Statistical Analysis

All data were analyzed using GraphPad Prism 9.0 software. One-way ANOVA was used for comparisons among two or more groups. *p* < 0.05 was considered statistically significant. All data were given as mean ± standard deviations (SD).

## 3. Results

### 3.1. Molecular Docking Analysis

**Figure 1:**
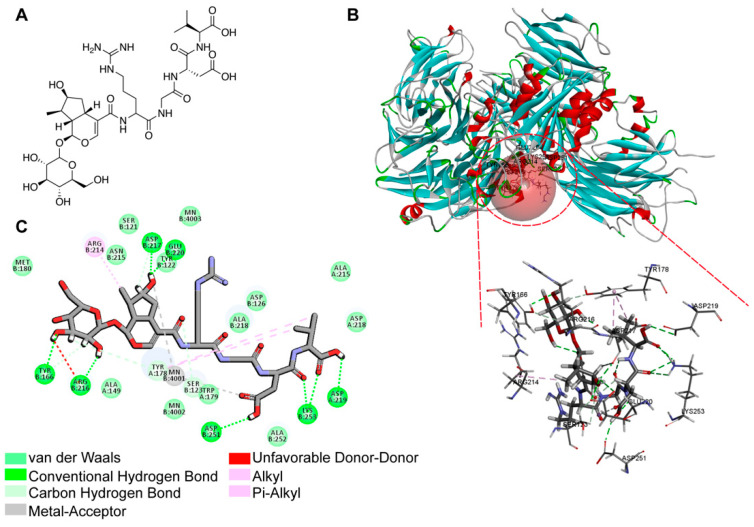
Molecular docking results. (**A**) The structure of compound LA-1. (**B**) Molecular docking and enlarged view of LA-1 and the active pocket of αvβ3 protein (PDB: 1L5G). (**C**) 2D diagram of the interaction force between LA-1 and αvβ3 protein.

Cyclic RGD-containing peptides are highly valuable ligands of αvβ3 integrin. Numerous RGD peptide analogues have been synthesized and subjected to targeting αvβ3 integrin [30,31]. In this study, we designed the compound loganin-RGD (Figure 1A), which uses RGDV as an αvβ3 integrin targeting motif. Initially, we assessed the binding affinity of LA-1 to αvβ3 integrin using CDOCKER (Figure 1B). As presented in Table 1, LA-1 exhibits high binding affinity to αvβ3 integrin. While the carboxyl hydrogen on compound LA-1 forms a hydrogen bond with aspartic acid (Asp) in the binding site of the αvβ3, the carbonyl oxygen on compound LA-1 coordinates with the manganese ion (Mn^2+^) (Figure 1C). Both types of interaction allow the compound to embed securely into the binding site [32], suggesting that LA-1 has potentials to target αvβ3 integrin.

### 3.2. Identification and Characterization of LA-1

**Figure 2:**
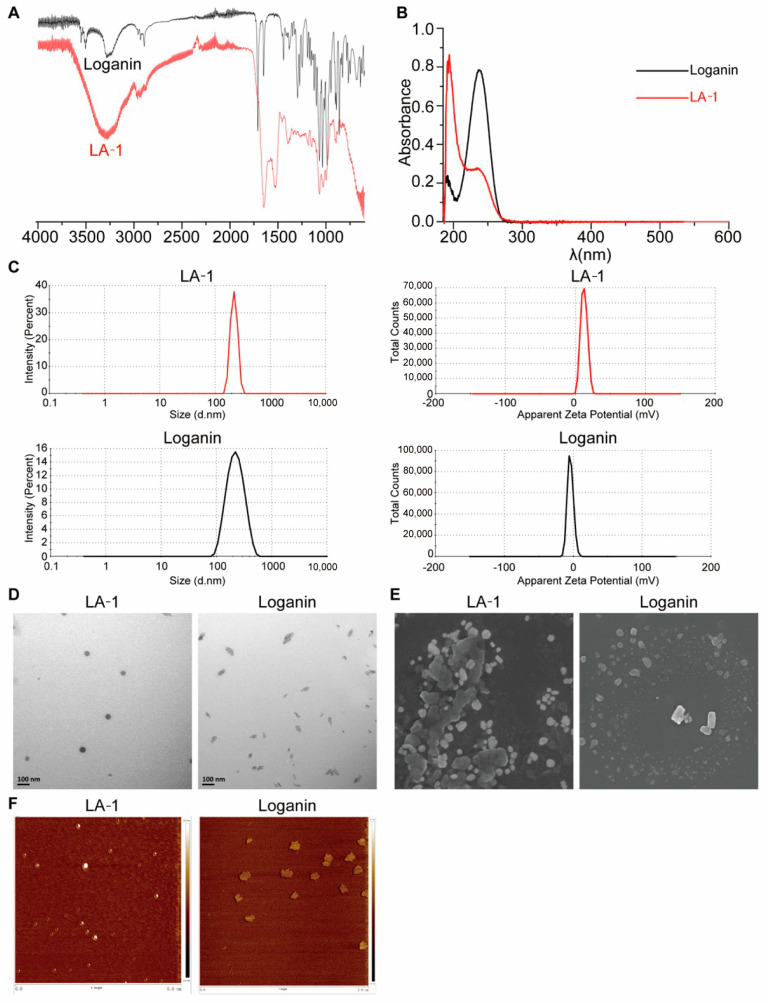
Characterization of LA−1 and loganin. (**A**) The FT−IR spectra of LA-1 and loganin. (**B**) The UV spectra of LA−1 and loganin. (**C**) Particle size distribution and zeta potential of LA−1 and loganin. (**D**) TEM images of LA−1 and loganin. (**E**) SEM images of LA−1 and loganin. (**F**) AFM images of e LA−1 and loganin.

LA-1 was synthesized via a condensation reaction and the product was identified using ^1^H NMR, ^13^C NMR, and FT-MS (Figures S1–S3). The purity of LA-1 was confirmed to exceed 98% by LC–MS (Figure S4). In our previous studies, we found that RGD-containing analogues could form nanoparticles via self-assembly [33,34]. Therefore, the nanoscale properties of LA-1 were also evaluated here. Initially, we measured the particle size and polydispersity index (PDI) of both LA-1 and loganin (Table 2 and Figure 2C). The average particle size of LA-1 was 219.9 ± 30.95 nm, with a PDI of 0.421 and a zeta potential of 12.2 ± 4.66 mV. In contrast, loganin exhibited an average particle size of 231.5 ± 81.235 nm, a PDI of 0.194, and a zeta potential of −4.16 ± 4.40 mV. The positive zeta potential of LA-1 indicates enhanced stability in suspension due to the repulsive forces between particles, reducing the likelihood of aggregation [35], which could also facilitate better interaction with negatively charged cell membranes, potentially enhancing cellular uptake [36].

As observed through different microscopic techniques (Figure 2D–F), LA-1 exhibited spherical and regular nanostructures, while the nanoparticles of loganin appeared irregular and unevenly dispersed. These observations indicate that LA-1 possesses superior nanoscale properties. In conclusion, the excellent physicochemical characteristics of LA-1 nanoparticles may enhance its performance in drug delivery.

### 3.3. Free Radical Scavenging and Cellular Uptake Assay

**Figure 3:**
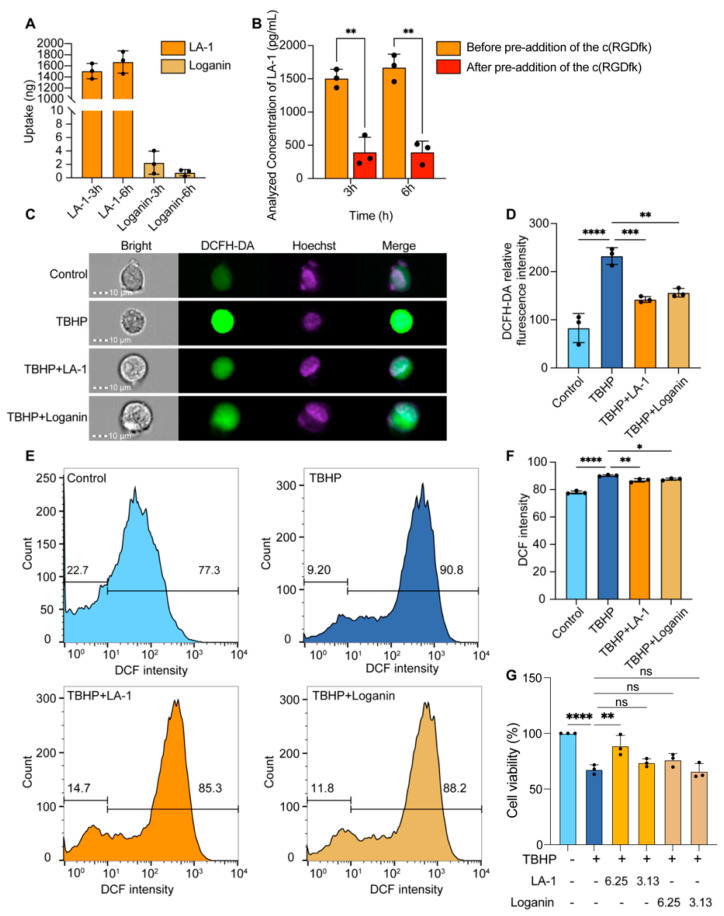
Evaluation of the neuroprotective and antioxidant effects of LA-1 and loganin. (**A**) Comparative Uptake of LA-1 and Loganin in BEnd.3 cells. (**B**) Drug of LA-1 uptake over time was measured by HPLC-MS after pre-addition of the αvβ3 inhibitor c(RGDfk). (**C**) Representative images of BEnd.3 cells stained with DCFH-DA (green) and Hoechst (purple) under different treatment groups. (**D**) Quantification of the percentage of DCFH-DA relative fluorescence intensity in each treatment group. (**E**) Flow cytometry analysis of DCFH-DA stained BEnd.3 cells to detect ROS levels under different treatment conditions. (**F**) Quantification of the percentage of DCFH-DA relative fluorescence intensity in each phase. (**G**) LA-1 and loganin’s protective effects against TBHP-induced cytotoxicity. Data were presented as mean ± SD, with statistical significance indicated as * *p* < 0.05, ** *p* < 0.01, and *** *p* < 0.001. **** *p* < 0.0001, ns represents not significant, n = 3.

As the active group of LA-1, loganin was reported to be able to reduce oxidative stress [19]. Therefore, we assessed the free radical scavenging activity of LA-1 using electron spin resonance (ESR). LA-1 significantly reduced the ESR signal intensity of NO, ·OH, and DPPH radicals (Figure S5), indicating the conjugation with RGDV may not affect the antioxidant capacity of loganin. To validate the integrin targeting ability of LA-1, an αvβ3 highly expressing cell line, BEnd.3, was used for uptake assay. Initially, the cytotoxicity of LA-1 and loganin on BEnd.3 cells was determined using the MTT assay. After 24 h treatment with LA-1 or loganin, no significant change in cell viability was observed from all three concentrations. MTT assay results indicated that LA-1 did not exhibit significant cytotoxicity under the given conditions (Figure S6). Then, the middle concentration in MTT assay (6.25 μM) was chosen for cellular uptake assay. BEnd.3 cells were incubated with 6.25 μM of LA-1 or loganin for 3 or 6 h, respectively. Intracellular LA-1 and loganin were then extracted and quantified by LC-MS/MS. The intracellular amount of loganin reached a plateau after 3 h of incubation, while LA-1 continued to accumulate, indicating that the modified LA-1 has enhanced cellular uptake efficiency compared to loganin. After 3 h of incubation, the cellular uptake of LA-1 was approximately 670-fold higher than that of loganin, and after 6 h of incubation, the uptake increased to approximately 2148-fold higher. This demonstrates that conjugation with RGDV significantly enhanced cellular uptake efficiency (Figure 3A). To validate the increase in cellular uptake of LA-1 is mediated by integrin αvβ3, we pretreated BEnd.3 cells with 100 μM of integrin αvβ3 inhibitor c(RGDfK) for 1 h, then performed cellular uptake assay as described above. As expected, the cellular uptake of LA-1 decreased significantly (by approximately 3-fold) with the presence of c(RGDfK) (Figure 3B).

### 3.4. The Antioxidant and Cell Protective Activity of LA-1 in an Oxidative Stress Cell Model

Neuroprotection of loganin is thought to be associated with its ROS scavenging activity [19]. To evaluate the cellular protective activity of LA-1, we stimulated BEnd.3 cells with 150 μM of TBHP for 2 h to induce oxidative stress after the different treatments were applied to cells. The levels of ROS generation were detected through 2,7-dichlorofluorescein diacetate (DCFH-DA) staining and measured with fluorescence intensity measured by single-cell imaging or flow cytometry. Compared with the non-treatment group, cells treated with 150 μM TBHP exhibited stronger green fluorescence (Figure 3C,D), indicating the successful establishment of the oxidative stress cell model. The cells pretreated with 6.25 μM LA-1 for 4 h exhibited a significant reduction in fluorescence intensity (from 232.303 to 142.152), which was much lower than that observed with loganin at the same concentration (Figure 3C,D). Quantitative analysis results from the flow cytometry assay also supported the above observations (Figure 3E,F), confirming that the ROS scavenging activity of LA-1 was enhanced compared to loganin, particularly when compared to the TBHP-damaged group.

Next, the protective effects of LA-1 against oxidative stress-induced cell death were also evaluated. The BEnd.3 cells were pre-treated with LA-1 or loganin for 24 h, followed by exposure to 200 μM of TBHP for 4 h. The pre-treatment with 6.25 μM of LA-1 significantly alleviated TBHP-induced cell death, but not with 6.25 μM of loganin (Figure 3G).

### 3.5. Blood–Brain Barrier Penetration of LA-1 After Ischemic Injury

**Figure 4:**
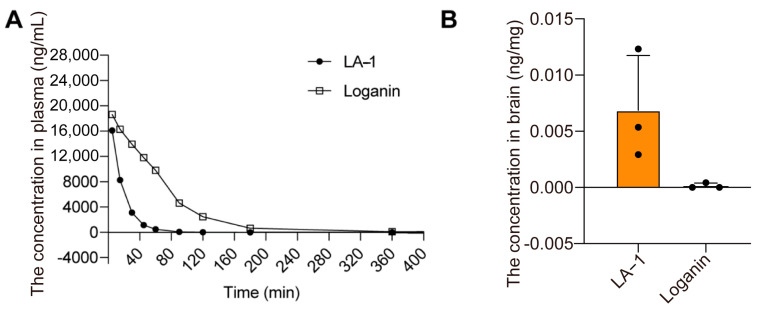
Pharmacokinetics and brain accumulation of LA-1 and loganin. (**A**) Pharmacokinetic analysis of LA-1 and loganin in plasma. (**B**) Quantification of LA-1 and loganin in ischemic brain tissue. Data were presented as the mean ± SD, n = 3.

Integrin αvβ3 mediated ischemic brain lesions targeting delivery have been demonstrated [26]. In this study, we performed a pharmacokinetic analysis on LA-1 and loganin using the LC–MS/MS method with an intravenous tail injection at 30 mg/kg. From the plasma drug concentration time curve, we observed that the half-life of LA-1 and loganin is around 10 or 45 min, respectively (Figure 4A). The plasma concentration of LA-1 was below the limit of detection (LOD) at 90 min, while loganin was undetectable at 180 min after infusion.

To detect the brain accumulation of LA-1 and loganin after ischemic injury, brain tissue from the rats which were subjected to induced ischemia was collected at 3 h after administration. In contrast to the plasma half-life, the brain accumulation of LA-1 was dramatically higher than that of loganin (Figure 4B). Considering the cellular uptake data, the above results suggest that LA-1 penetrates the brain much more rapidly than that of loganin itself. This also means targeting at αvβ3 is a successful drug delivery strategy to the brain.

### 3.6. LA-1 Alleviates Brain Ischemia–Reperfusion Injury

**Figure 5:**
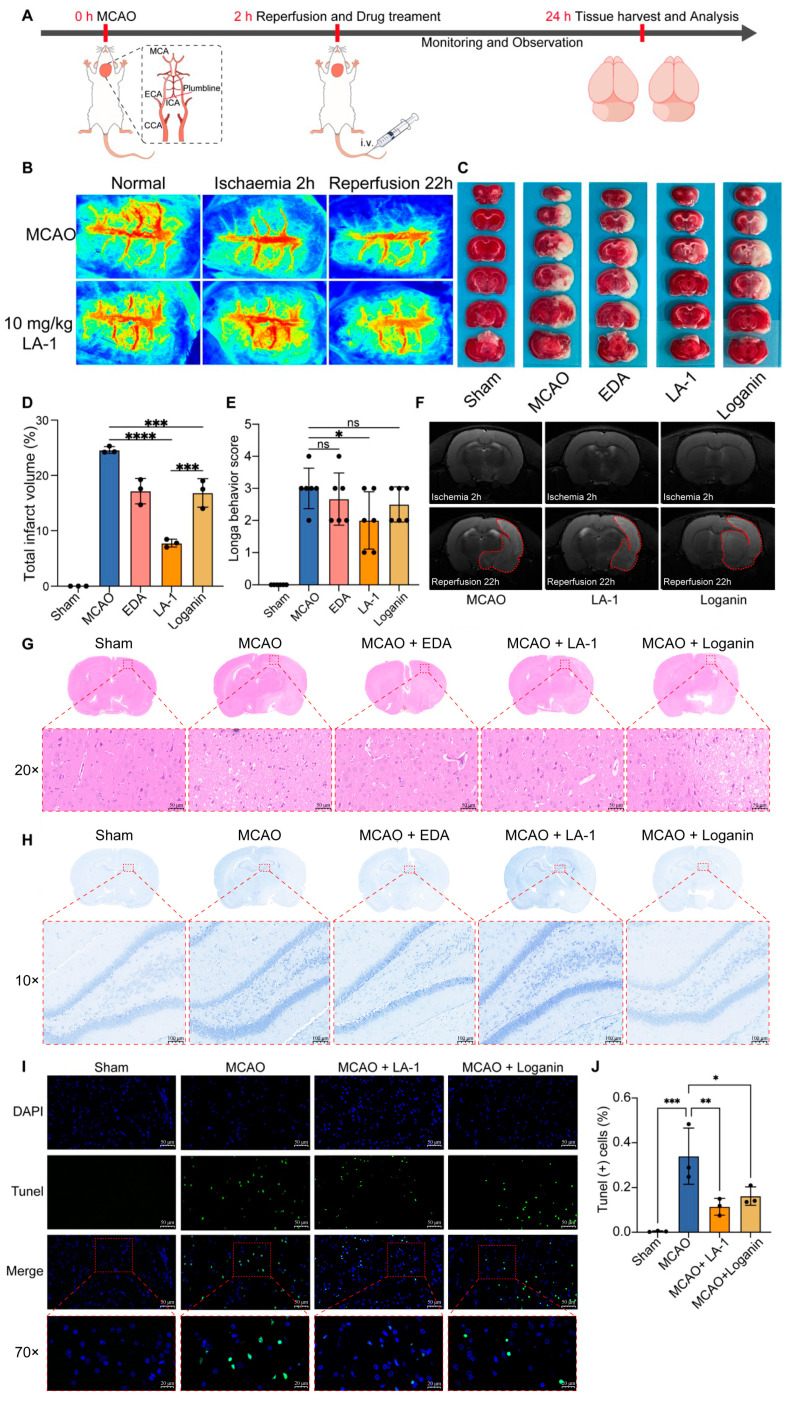
Therapeutic effect of the LA-1 on a rat model of cerebral ischemia/reperfusion injury. (**A**) The experimental timeline for in vivo evaluation of LA-1 and loganin. (**B**) Non-invasive, trans-cranial laser Doppler perfusion imaging to verify occlusion at 2h,22h after MCAO. (**C**) Representative images of TTC-stained brain slices after treatment with LA-1 and loganin for 22 h in MCAO rat model. (**D**) Corresponding infarct size statistics. (**E**) Neurological scores of MCAO rats after treatment with LA-1 and loganin for 22 h. (**F**) MRI imaging of MCAO rats before and after the i.v. injection of the LA-1 and loganin. (**G**) H&E staining, scale bar = 50 μm. (**H**) Nissl staining, scale bar = 100 μm and (**I**) tunel staining, scale bar = 100 μm, scale bar = 20 μm of brain tissues from different treatment groups. (**J**) Recorded the number of tunel cells by image J. Data were presented as the mean ± SD, * *p* < 0.05, ** *p* < 0.01, and *** *p* < 0.001. **** *p* < 0.0001, ns represents not significan.

To investigate the effects of LA-1 on acute brain ischemia–reperfusion injury, we established a brain ischemia–reperfusion injury model in adult rats (Figure 5A). Following a 2 h induction with ischemia, we monitored cerebral blood flow dynamics using a laser speckle flow imaging system. The results showed a significant decrease in cerebral blood flow at the ischemic brain lesion in the MCAO model (Figure 5B), confirming the successful induction of the stroke model. After the occluding filament was removed, different treatments were administered to the MCAO rats via tail vein injection. Notably, treatment with LA-1 resulted in a marked increase in cerebral blood flow compared to the model group, highlighting the therapeutic effect of LA-1 in restoring blood flow.

By performing magnetic resonance imaging analysis, we found that the brain damage was not prominent after 2 h of ischemia but was enhanced after 22 h of reperfusion (Figure 5F). Therefore, we evaluated the neurological function of MCAO model animals using the Zelonga score at 22 h post-treatment (Figure 5E). Most rats in the model group had a neurological score of 3, indicating worsening neurological deficits after MCAO surgery. The rats treated with 3 mg/kg of edaravone or 30 mg/kg loganin showed a reduction trend in neurological score but without statistically significant difference. In contrast, the treatment with 10 mg/kg of LA-1 reduced the scores significantly, suggesting that LA-1 may improve the neurological functions of rats after ischemia–reperfusion injury.

TTC staining displayed a significant infarct area reduction in both edaravone and loganin treatment groups, consistent with the published data from other researchers [17,37,38]. In comparison with loganin, the infarct area in LA-1 group is more than 2-fold less than that of loganin group (7.743% vs. 16.843%), indicating that the neuroprotective effect of 10 mg/kg LA-1 is more potent than that of 30 mg/kg loganin. To evaluate the effects of LA-1 and Loganin on neuronal damage after cerebral ischemia–reperfusion, we performed H&E and Nissl staining (Figure 5G,H). In the H&E staining (Figure 5G), cortical neurons in the MCAO group showed significant damage, with disorganized arrangement and poor cellular structure compared to the sham group. LA-1 treatment improved these changes, restoring the regular arrangement and clearer structure of neurons. Loganin treatment also improved the morphology, though to a lesser extent than LA-1. Nissl staining (Figure 5H) revealed similar patterns, with the MCAO group displaying fewer neurons and weaker staining, indicating damage. LA-1 treatment significantly increased neuron count and restored Nissl substance staining, suggesting better neuroprotection. While Loganin also showed improvement, the recovery was less pronounced compared to LA-1. TUNEL staining (Figure 5I) assay displayed stronger green fluorescence in the MCAO group compared to the sham surgery group, indicating neuronal apoptosis was significantly induced by cerebral ischemia–reperfusion. When treated with LA-1, the green fluorescence-positive neurons significantly decreased from 34.0% to 11.4% (Figure 5J). The staining results suggest LA-1 effectively inhibits cerebral ischemia–reperfusion-induced neuron death, which is consistent with the reduction in neurological score. Taken together, LA-1 treatment may alleviate brain ischemia–reperfusion injury in the MCAO rat model. Even at one-third dose of loganin, the effectiveness of LA-1 is much stronger than that of loganin, indicating the modification with αvβ3 targeting dramatically improved the potency of loganin.

Additionally, tissues from each group were also evaluated by performing H&E staining. No significant pathophysiological changes were observed in the heart, liver, spleen, and kidney tissue sections (Figure S7), indicating LA-1 may not lead to acute toxicity in vivo.

### 3.7. LA-1 Alleviates Oxidative Stress Damage and Inflammation Induced by Cerebral Ischemia–Reperfusion Injury

**Figure 6:**
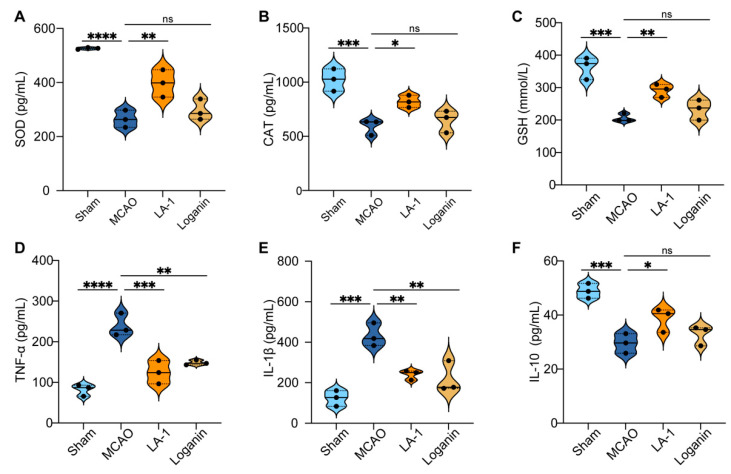
LA-1 modulates antioxidant enzyme levels and inflammatory cytokine expression in rat model of cerebral ischemia/reperfusion injury. Expression levels were shown as (**A**) superoxide dismutase (SOD), (**B**) catalase (CAT), (**C**) glutathione peroxidase (GSH-Px), (**D**) TNF-α, (**E**) IL-1β, and (**F**) IL-10 in the brain tissue of different treatment groups. Data were presented as the mean ± SD, * *p* < 0.05, ** *p* < 0.01, and *** *p* < 0.001. **** *p* < 0.0001, ns represents not significant, n = 3.

Both oxidative stress and inflammation contribute to the neurological deficit induced by cerebral ischemia–reperfusion. To further investigate the neuroprotective mechanisms of LA-1, we evaluated its antioxidant activity by measuring the levels of superoxide dismutase (SOD), catalase (CAT), and glutathione peroxidase (GSH-Px) in brain tissue. The results exhibit that ischemia–reperfusion injury (MCAO group) led to decreased levels of SOD, CAT, and GSH. Compared to the MCAO group, the LA-1 treatment restored the levels of SOD, CAT, and GSH (Figure 6A–C), confirming the antioxidant activity LA-1 in vivo. We next assayed the anti-inflammation activity of LA-1 by measuring the levels of TNF-α, IL-1β, and IL-10 in brain tissue. Compared to the sham group, the MCAO group exhibited increased levels of the pro-inflammatory factors TNF-α and IL-1β, and decreased levels of IL-10. After treatment with LA-1, the levels of TNF-α and IL-1β were significantly reduced, while the levels of IL-10 increased (Figure 6D–F). These results indicate that LA-1 can effectively reduce inflammation and oxidative stress damage induced by cerebral ischemia–reperfusion injury in vivo.

### 3.8. Proteomic Analysis of Potential Signaling Pathways in MCAO Rats Treated with LA-1

**Figure 7:**
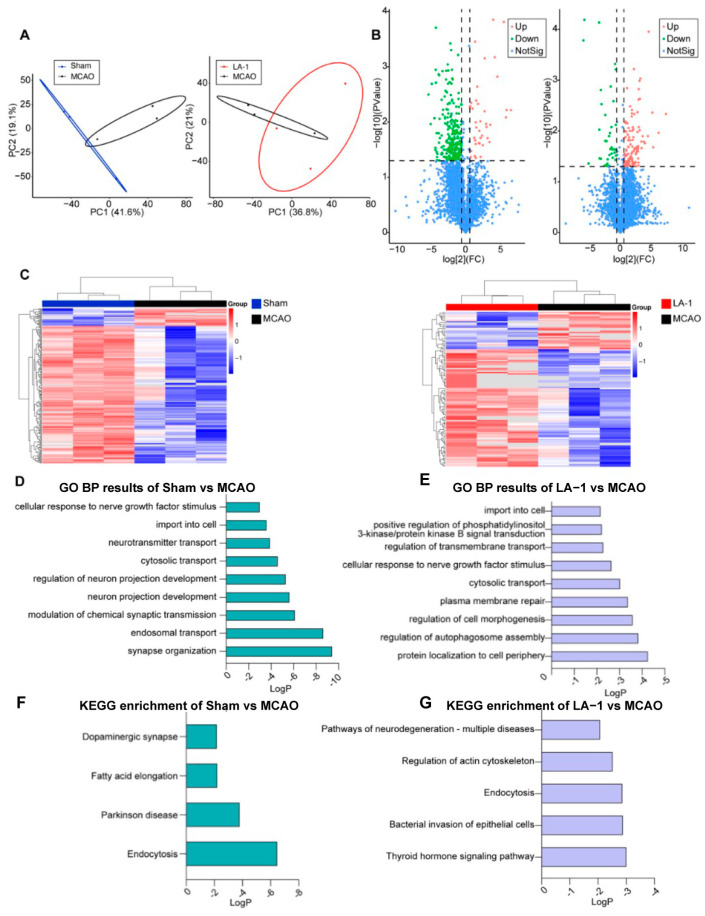
Identification of differentially expressed proteins from ischemic penumbra. (**A**) PCA of gene expression in Sham, MCAO, and LA-1-treated groups. (**B**) The proteome profiles in MCAO group compared with the Sham group, LA-1-treated group compared with the MCAO group. (**C**) The heatmap of DEPs in MCAO group compared with the Sham group, LA-1-treated group compared with the MCAO group, and the differential protein overlap: Sham vs. MCAO and MCAO vs. LA-1. (**D**) GO BP results of DEPs in MCAO group compared with the Sham group. (**E**) GO BP results of DEPs in LA-1-treated group compared with the MCAO group. (**F**) KEGG enrichment of DEPs in MCAO group compared with the Sham group. (**G**) KEGG enrichment of DEPs in LA-1-treated group compared with the MCAO group.

To further investigate the molecular mechanisms underlying the neuroprotective effects of LA-1 in the MCAO model, we performed proteomic analysis on the ischemic side (right hemisphere) of brain tissues, both with and without LA-1 treatment. After rigorous data filtering to eliminate low-scoring spectra and removal of redundant proteins, a total of 4143 proteins were identified and quantified. Principal Component Analysis (PCA) on the proteomic data revealed distinct protein expression patterns among the different groups (Figure 7A). After differential expression analysis, proteins meeting the criteria of *p* < 0.05 and Fold change > 1.5 were considered differentially expressed proteins (Figure 7B). Compared to the sham group, 311 differentially expressed proteins were identified from the MCAO group, including the protein levels of 40 were increased and 272 were decreased. Between the two cerebral ischemia–reperfusion groups, LA-1 treatment resulted in the protein levels of 122 increased and 38 decreased when compared to the non-treatment group. Heat map displayed the normalized abundance of identified differential proteins (Figure 7C). We further screened out genes without corresponding protein names and performed a Venn plot. The Venn diagram illustrates the overlap of differentially expressed proteins between the comparisons: sham vs. MCAO and MCAO vs. LA-1. A total of 44 overlapping differential proteins were identified from both comparisons.

To reveal the potential cellular pathways involved in cerebral ischemia–reperfusion injury, we performed functional enrichment analysis on the identified differential proteins from each group (Figure 7D,E). The results from Gene Ontology (GO) enrichment analysis displayed that the proteins affected by ischemia–reperfusion are involved in synapse functions, neuron projection and intracellular transport (Figure 7D), while the proteins regulated by LA-1 are involved in PI3K and autophagy related pathways (Figure 7E). Kyoto Encyclopedia of Genes and Genomes (KEGG) pathway annotation analyses revealed that endocytosis and Parkinson disease related pathways are associated with ischemia–reperfusion (Figure 7F) while the proteins regulated by LA-1 may participate in neurodegeneration-multiple diseases (Figure 7G).

### 3.9. LA-1 Alleviates MCAO Damage Through the PI3K-Akt and Nrf2/HO-1 Pathways

**Figure 8:**
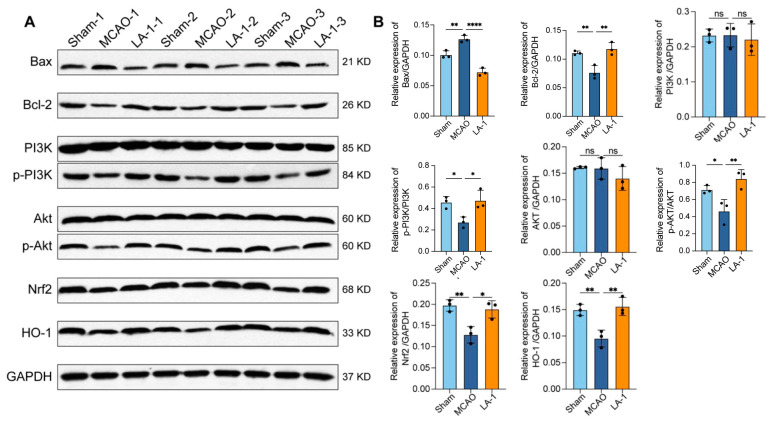
WB analysis of brain tissue (n = 3) (**A**) Representative Western blot images displaying the bands corresponding to Bax, Bcl-2, PI3K, p-PI3K, Akt, p-Akt, Nrf2, HO-1, GAPDH. The samples corresponded to brain tissue in all cases. (**B**) Quantification of band densities for Bax/GAPDH, Bcl-2/GAPDH, PI3K/GAPDH, p-PI3K/PI3K, AKT/GAPDH, p-AKT/AKT, Nrf2/GAPDH, HO-1/GAPDH. Data were presented as the mean ± SD, * *p* < 0.05, ** *p* < 0.01, **** *p* < 0.0001, ns represents not significant, n = 3.

Based on our proteomic data, we examined the levels of proteins associated with PI3K-Akt and Nrf2/HO-1 pathways by Western blot (Figure 8A,B). Compared to sham group, the MCAO group exhibited significantly decreased levels of phosphorylated PI3K (p-PI3K), phosphorylated Akt (p-Akt), Nrf2, and HO-1, indicating that both PI3K-Akt and Nrf2/HO-1 pathways were inhibited during cerebral ischemia–reperfusion injury. In addition, the levels of pro-apoptosis protein Bax were elevated, while the levels of anti-apoptosis protein Bcl-2 were reduced significantly in MCAO group. After treatment with 10 mg/kg of LA-1, the abundance of all the proteins we detected was restored to normal levels. These findings strongly suggest that LA-1 mitigates MCAO-induced damage by activating the PI3K-Akt and Nrf2/HO-1 signaling pathways, and inhibiting the apoptosis pathway.

## 4. Discussion

In recent years, targeted nanomedicine has emerged as an effective treatment modality for delivering drugs to the site of cerebral ischemia [39]. Various receptors and transporters are highly expressed on brain capillary endothelial cells to facilitate the transport of nutrients [40]. By modifying drugs with ligands of these receptors or transporters, significant increases in brain drug accumulation were observed.

It was demonstrated that integrin αvβ3 was initially detected in the ischemic area of the brain 2 h after middle cerebral artery occlusion (MCAO) and is highly expressed thereafter [22,41]. In a transient MCAO mouse model, exosomes modified with c(RGDyK) significantly accumulated in the brain lesion area. Further experiments revealed that the targeting ability of cRGD-Exosome was mediated by the recognition of αvβ3, which was induced expressing on brain vascular endothelial cells by ischemia [42]. In another study, bLXW7, a ligand of integrin αvβ3, modified nanoparticle CeNPs exhibited effective accumulation in the peri-infarct brain region, and protected neurons from acute cerebral ischemia–reperfusion injury [43]. These findings suggest that integrin αvβ3 ligands have inherent advantages in delivering drugs to cerebral ischemic sites.

In this study, we designed an αvβ3 targeting agent LA-1 by conjugating a neuroprotective molecule loganin with one RGD-containing peptide for neuroprotection against cerebral ischemia–reperfusion injury. Our data demonstrated that LA-1 penetrated BEnd.3 cells rapidly through αvβ3 mediated cellular uptake, leading to the intracellular amount of LA-1 was approximately 500-fold higher than that of loganin after 3 h of incubation. In vivo, although the plasma half-life of LA-1 is shorter than that of loganin, we still detected the dramatically higher brain accumulation level of LA-1. Both in vitro and in vivo results supported our hypothesis that conjugation with αvβ3 ligands increases active molecules’ brain delivery.

As the active group of LA-1, loganin was reported to protect neurons from cerebral ischemia–reperfusion injury via reducing oxidative stress and anti-inflammation [19]. The antioxidant activity of LA-1 was validated through free radical scavenging and TBHP-induced ROS generation assays. Compared with loganin, the activity of LA-1 did not significantly increase in inhibiting TBHP-induced ROS generation in BEnd.3 cells. This observation conflicted with the result from the cellular uptake assay, indicating structure modification on the selected site may decrease the antioxidant activity of loganin in vitro. In addition, LA-1 effectively protected BEnd.3 cells from TBHP-induced cell death at the given condition, but not loganin. This finding supported the potential of LA-1 against cerebral ischemia–reperfusion injury and suggested that reducing oxidative stress may not be the only mechanism of cell protection of LA-1.

Using the MCAO model, we evaluated the neuroprotective activity of LA-1 against cerebral ischemia—reperfusion injury. Intravenous injection with LA-1 effectively restored cerebral blood flow and significantly improved neurological deficit scores in MCAO rats. TTC staining and pathology sections displayed LA-1 treatment reduced infarct volume, restored the ischemia–reperfusion injury induced morphology changes of the cortical and hippocampal zone, and inhibited neuron apoptosis. These data confirmed the therapeutic potential of LA-1 against cerebral ischemia–reperfusion injury.

We propose that the neuroprotective effect of LA-1 is achieved through a dual mechanism. On one hand, LA-1 enters brain endothelial cells via the αvβ3 integrin receptor, reducing ROS levels within these cells, thereby indirectly protecting the cerebrovascular system and neurons. On the other hand, after crossing the blood–brain barrier, LA-1 acts directly on neurons by scavenging intracellular ROS and restoring the expression of antioxidant enzymes such as SOD, CAT, and GSH, ultimately inhibiting ROS-induced neuronal apoptosis.

This dual-action mechanism explains the neuroprotective effects of LA-1 in ischemia–reperfusion injury. It not only demonstrates LA-1’s capacity to clear ROS in endothelial cells but also emphasizes its direct antioxidant and anti-apoptotic effects on neurons. Overall, the neuroprotective effects of LA-1 are achieved by reducing oxidative stress, suppressing inflammatory responses, and preventing neuronal death through multiple pathways.

During cerebral ischemia and reperfusion, the increased release of arachidonic acid results in an accumulation of lipid peroxides [44], while the intracellular calcium overload eliminates mitochondrial oxidative phosphorylation, reduces mitochondrial membrane potential, and causes excessive reactive oxygen species (ROS) generation [45]. The excessive production of lipid peroxides and ROS exacerbates oxidative stress responses, which in turn induce neuronal apoptosis [46]. Endogenous antioxidant mechanisms play a balancing role, and it is essential to scavenge ROS by upregulating antioxidant enzymes [47]. Nrf2 is considered a major regulator of redox homeostasis [48], regulating antioxidant and anti-inflammatory responses [49]. Under oxidative stress, activated Nrf2 binds to antioxidant response elements (AREs) to promote detoxification and the production of antioxidant enzymes such as HO-1, NQO1, and GSH [50]. Previous studies have shown that overexpression of HO-1 significantly reduces cerebral infarction size in permanent MCAO experiments [51]. We detected significant decreases in antioxidant enzymes, such as SOD, CAT, and GSH, and the reduced activity of the Nrf2/HO-1 signaling pathway in MCAO rat brains. After treatment with LA-1, the levels of those dysregulated proteins and the activity of the Nrf2/HO-1 signaling pathway were partly restored, indicating the mechanism of neuroprotection of LA-1 may be associated with oxidative stress response pathways.

Both the increased release of arachidonic acid and the intracellular calcium overload are associated with the reduced activity of WNT signaling pathway during cerebral ischemia–reperfusion injury [52] Inhibition of Wnt/β-catenin signaling was reported in ischemic stroke patients and corresponding mouse models, which is considered to contribute to the release of inflammatory factors TNF-α, IL-1, IL-6,and IL-8, and aggravates the inflammatory response [53]. Our study also observed increases of pro-inflammation cytokines TNF-α and IL-1β in MCAO rat brains. Similar to antioxidant enzymes, the increases of pro-inflammation cytokines were reversed by LA-1 treatment. Therefore, we hypothesized that regulation of WNT signaling pathway may play an essential role in the neuroprotection of LA-1 against cerebral ischemia–reperfusion injury.

By performing proteomic analysis, we found that PI3K associated pathways may be involved in cerebral ischemia–reperfusion injury. PI3K/Akt regulates pathological and physiological processes such as cell apoptosis [54], neuroinflammation, and oxidative stress [55]. Activation of the PI3K/Akt signaling pathway was reported to reduce apoptosis by enhancing Bcl-2 expression in brain ischemia–reperfusion injury [56]. Furthermore, the PI3K/Akt/Wnt signaling crosstalk was identified as a critical pathological process in brain ischemia–reperfusion injury. As the PI3K/AKT signaling pathway is positioned upstream of Wnt/β-catenin signaling, activation of PI3K/Akt signaling pathway simultaneously activates Wnt/β-catenin signaling [52]. To validate the mechanisms of neuroprotection of LA-1, we detected the activation of PI3K/Akt pathway by Western blot. Our results revealed that LA-1 restored the activation of PI3K/Akt pathway, whose functions were inhibited in ischemia–reperfusion injury. In addition, the activation of PI3K/Akt pathway by LA-1 treatment is accompanied by the downregulation of Bax and upregulation of Bcl-2. These data further supported that LA-1 protects neurons from cerebral ischemia–reperfusion injury via activating PI3K/Akt pathway.

Taken together, we considered that the neuroprotection of LA-1 against cerebral ischemia–reperfusion injury is worked by activating PI3K/Akt pathway. The activation of PI3K/Akt pathway probably restored the activity of WNT signaling pathway, which inhibited the excessive production of ROS and inflammation and subsequently prevented neurons from ischemia–reperfusion injury induced apoptosis.

Despite our promising results, several limitations in our study need to be addressed. First, the plasma half-life of LA-1 is too short, which may limit its maximum brain accumulation and treatment efficacy. Second, we need to investigate the optimal dosage and administration regimen of LA-1 to further increase its efficacy under different pathological conditions. Lastly, the potential long-term side effects and safety of prolonged LA-1 usage require thorough investigation.

## 5. Conclusions

In summary, our results suggest that LA-1 accumulates rapidly in the ischemic brain lesions by targeting integrin αvβ3. Thus, it effectively alleviates cerebral ischemia–reperfusion injury in MCAO rats. The mechanisms of action of LA-1 are associated with suppressing inflammation and oxidative stress, as well as modulating the PI3K/Akt and Nrf2 signaling pathways. These findings highlight the potential of LA-1 as a therapeutic agent for cerebral ischemia–reperfusion injury, and the promising role of αvβ3 mediated cellular uptake for efficient drug brain delivery.

## Figures and Tables

**Table 1:** Molecular docking of compounds towards the active site of αvβ3 protein (PBD: 1L5G).

Compounds	CDOCKER Interaction Energy (Kcal/mol)
LA-1	83.5663
RGDV	73.9002
Loganin	49.9485

**Table 2:** Physicochemical characterization of LA-1 and loganin.

Groups	Particle Size (nm)	Zeta Potential (mV)
LA-1	219.9 ± 30.95	12.2 ± 4.66
Loganin	231.5 ± 81.23	−4.16 ± 4.40

## Data Availability

Data will be made available upon request.

## Supplementary Materials

The following supporting information can be downloaded at: https://www.mdpi.com/article/10.3390/antiox13111339/s1. Scheme S1. Preparation of LA-1. (i) HBTU, NMM and DMF; (ii) H_2_, Pd/C and CH_3_OH. Figure S1. ^1^ H NMR (300 MHz, DMSO-*d6*) spectra of LA-1. Figure S2. ^13^C NMR (75 MHz, DMSO-*d6*) spectra of LA-1. Figure S3. The mass spectrum of LA-1 (ES^+^). Figure S4. The purity of the LA-1. Figure S5. The ESR signal intensity of NO, ·OH, and DPPH radicals. Figure S6. The MTT assay result of LA-1 and loganin. Figure S7. Assessment of the biosafety of drugs in vivo. Table S1. Details of the gradient.
