# Supplementary material for: An RGD-Conjugated Prodrug Nanoparticle with Blood–Brain–Barrier Penetrability for Neuroprotection Against Cerebral Ischemia–Reperfusion Injury
Source: Antioxidants (Basel). 2024 Nov 1;13(11):1339. doi: 10.3390/antiox13111339 (PMC11591307; doi:10.3390/antiox13111339)
Supplement: Supplementary file 1 [file antioxidants-13-01339-s001.zip › SI文件.pdf]

**Supplementary Information for**

# An RGD-conjugated prodrug nanoparticle with blood-brain-barrier penetrability for Neuroprotection Against Cerebral Ischemia-Reperfusion Injury

Ayijiang Taledaohan[a,b], Maer Maer TuoHan[a,b,], Renbo Jia[a,b,], Kai Wang[a,b,], Liujia Chan[a,b,], Yijiang Jia[a,b,],Feng Wang[a,b,*], Yuji Wang[a,b,*]

[a]*Department of Medicinal Chemistry, College of Pharmaceutical Sciences of Capital Medical University, Beijing 100069, China*
[b]*Beijing Area Major Laboratory of Peptide and Small Molecular Drugs, Engineering Research Center of Endogenous Prophylactic of Ministry of Education of China, Beijing Laboratory of Biomedical Materials, Beijing 100069, China*
*Corresponding author:
Tel./fax: + 86 10 83911530.
E-mail address: wangyuji@ccmu.edu.cn (Yuji Wang).
*Corresponding author:
Email address: fengwang@ccmu.edu.cn (Feng Wang).

## 1. **Synthesis and identification of LA-1**

### 1.1 Materials

Loganic acid was obtained from Chengdu Push Bio-technology (Chengdu, P. R. China). 2-(1H-Benzotriazole-1-yl)-1,1,3,3-tetramethyluronium hexafluorophosphate (HBTU) and palladiu m carbon (Pb/C) were obtained from Shanghai Aladdin Biochemical Technology Co.

### 1.2 Synthesis of LA-1

Loganic acid (1 g) was dissolved by stirring with appropriate amount of N,N-Dimethylfor mamide (DMF), and 2 g of Arg($NO_2$)-Gly-Asp(OBzl)-Val-OBzl was added under an ice-w ater bath, and the pH was adjusted to 10 with N-Methylmorpholine (NMM). Room temper ature was stirred for 12 hours, and TLC (EA:$H_2O$:HAc=40:10:1) showed that loganin acid disappeared, concentrated to dryness under reduced pressure, purified by C18 column chro matography, and lyophilized to obtain LA-1. Yield:14.2%. $^1$H NMR (800 MHz, DMSO-d6) δ 10.11 (brs, 1H), 8.87 (brs, 1H), 8.49 (brs, 1H), 8.16 (s, 1H), 7.50 (d, J = 7.5 Hz, 1

H), 7.10 (d, J = 1.4 Hz, 1H), 7.07 (d, J = 8.6 Hz, 1H), 6.98 (brs, 2H), 5.07 (d, J = 4.9 Hz, 1H), 4.94 (brs, 2H), 4.53 (brs, 1H), 4.48 (d, J = 7.9 Hz, 1H), 4.36 (dt, J = 8.9, 5.0 Hz, 1H), 4.31 – 4.25 (m, 1H), 4.02 (dd, J = 8.6, 5.2 Hz, 1H), 3.88 (tt, J = 8.3, 5.5 Hz, 2H), 3.67 (dd, J = 11.9, 2.1 Hz, 1H), 3.58 (dd, J = 16.3, 4.2 Hz, 1H), 3.43 (dd, J = 11. 8, 6.4 Hz, 1H), 3.21 – 3.17 (m, 1H), 3.17 – 3.10 (m, 3H), 3.04 (t, J = 9.2 Hz, 1H), 2.9 8 (t, J = 8.4 Hz, 1H), 2.96 – 2.92 (m, 1H), 2.57 (dd, J = 16.2, 4.8 Hz, 1H), 2.34 (dd, J = 15.6, 3.1 Hz, 1H), 2.12 – 2.06 (m, 1H), 2.04 – 1.98 (m, 2H), 1.84 (td, J = 8.9, 4.9 Hz, 1H), 1.74 – 1.70 (m, 1H), 1.69 – 1.64 (m, 1H), 1.62 – 1.54 (m, 1H), 1.50 – 1.43 (m, 1H), 1.39 (ddd, J = 13.0, 7.9, 5.0 Hz, 1H), 0.99 (d, J = 6.9 Hz, 3H), 0.83 (d, J = 6.9 Hz, 6H). $^{13}$C NMR (201 MHz, DMSO) δ 173.07, 172.80, 170.41, 168.70, 166.60, 163. 33, 157.25, 145.24, 115.56, 98.50, 95.53, 77.27, 76.84, 73.15, 72.16, 70.08, 61.12, 57.42, 52.64, 49.96, 44.88, 42.32, 41.31, 40.53, 40.48, 37.09, 30.55, 30.52, 29.61, 25.01, 19.05, 1 7.82, 13.45. ESI$^+$-MS (m/e): 804.5[M+H]$^+$. $^1$H NMR and $^{13}$C NMR are shown in the Sup porting Information S1 and S2. The mass spectrum of LA-1 was shown in Supporting Inf ormation S3.

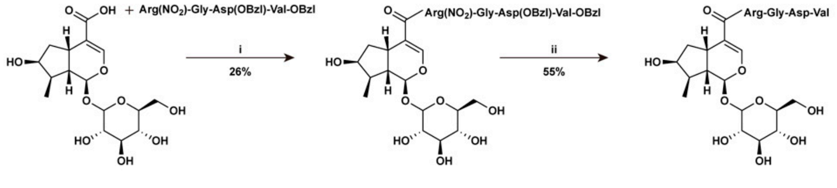


**Scheme 1** Preparation of LA-1. (i) HBTU, NMM and DMF; (ii) $H_2$, Pd/C and $CH_3OH$.

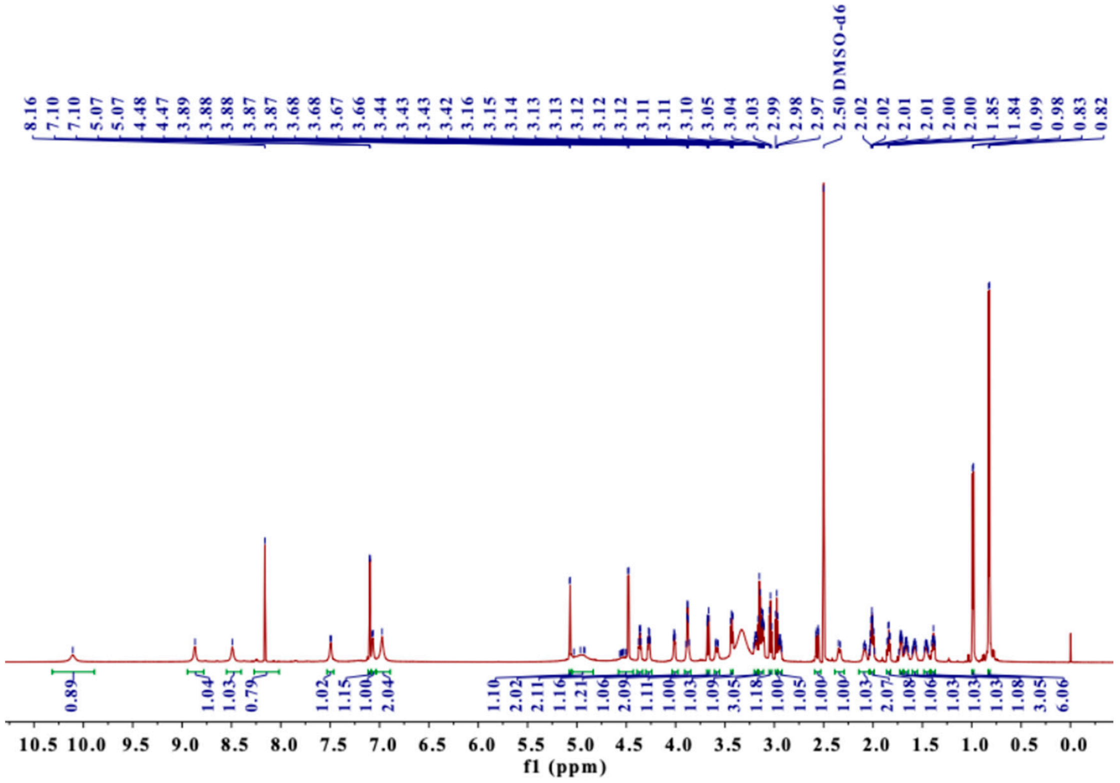


Figure S1 $^{1}$H NMR (300 MHz, DMSO-*d6*) spectra of LA-1

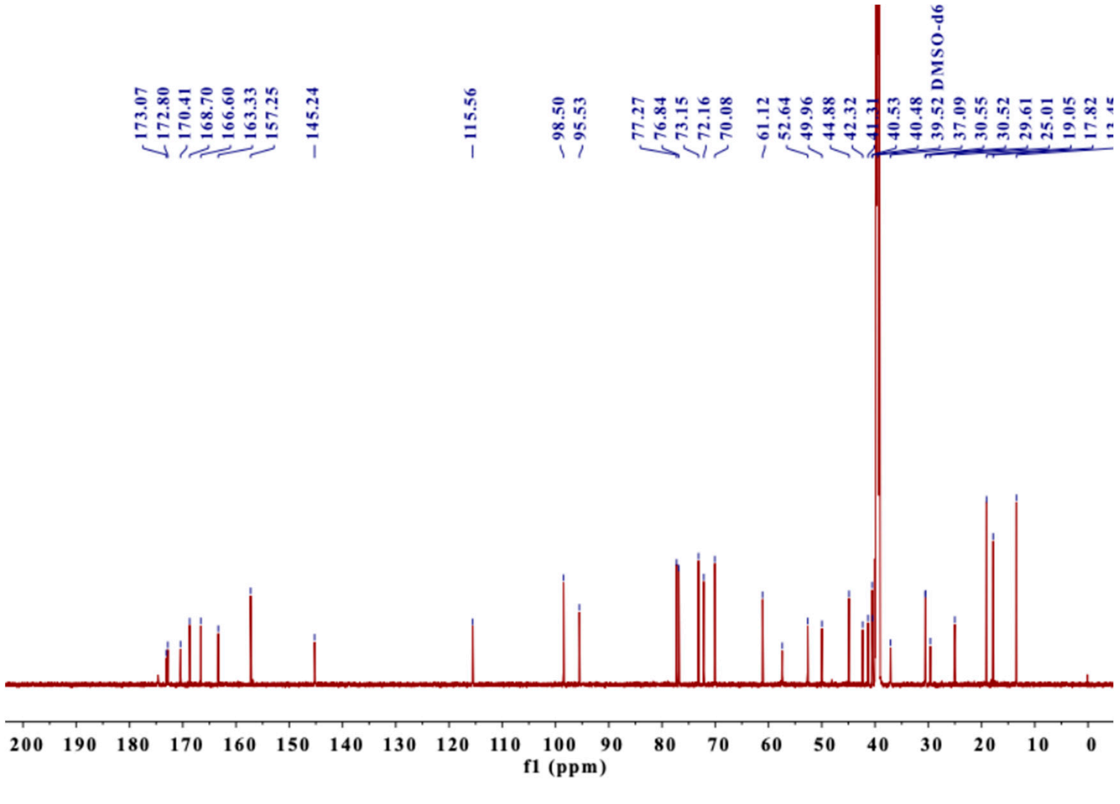


Figure S2 $^{13}$C NMR (75 MHz, DMSO-*d6*) spectra of LA-1

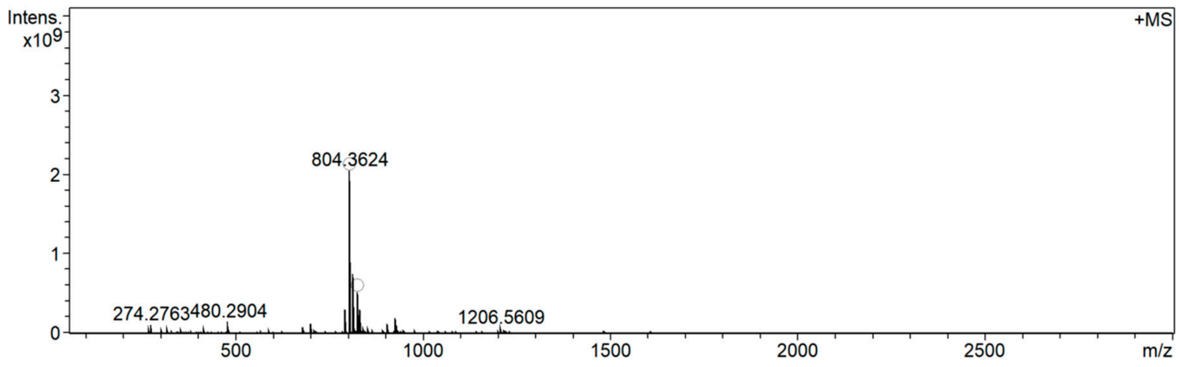


Figure S3 The mass spectrum of LA-1 ($ES^+$)

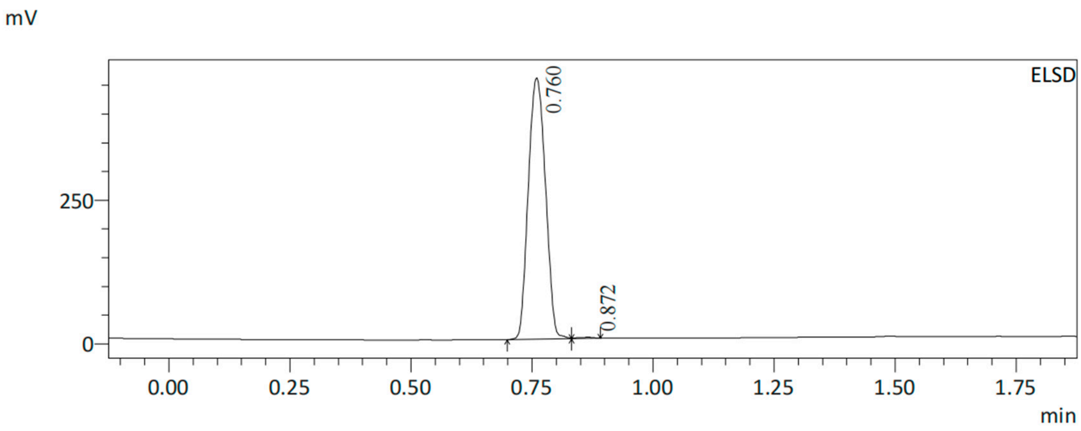


Peak Table

ELSD

| Peak# | Ret. Time | Height | Height% | Area | Area% |
|---|---|---|---|---|---|
| 6 | 0.760 | 454224 | 99.642 | 1124403 | 99.626 |
| 7 | 0.872 | 1632 | 0.358 | 4220 | 0.374 |
| Total | | 455856 | 100.000 | 1128624 | 100.000 |

Figure S4 The purity of the LA-1

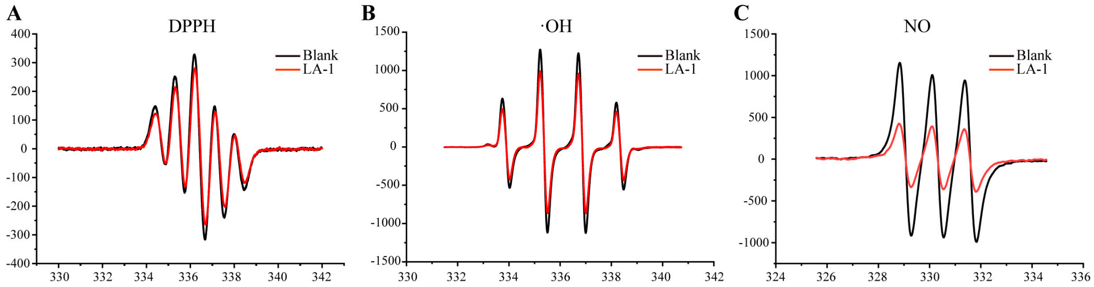


Figure S5 The ESR signal intensity of NO, ·OH, and DPPH radicals

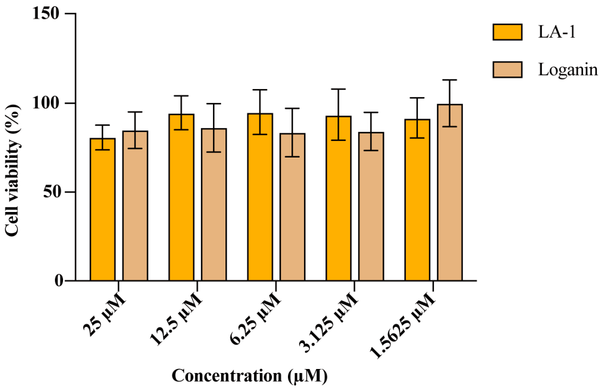


Figure S6 The MTT assay result of LA-1 and loganin

Table S1 Details of the gradient

| Time [mm:ss] | Duration [mm:ss] | Flow[nl/min] | Mixture[%B] |
|---|---|---|---|
| 00:00 | 00:00 | 700 | 1 |
| 01:00 | 01:00 | 700 | 9 |
| 101:00 | 100:00 | 700 | 28 |
| 113:00 | 12:00 | 700 | 40 |
| 116:00 | 03:00 | 700 | 99 |
| 120:00 | 04:00 | 700 | 99 |

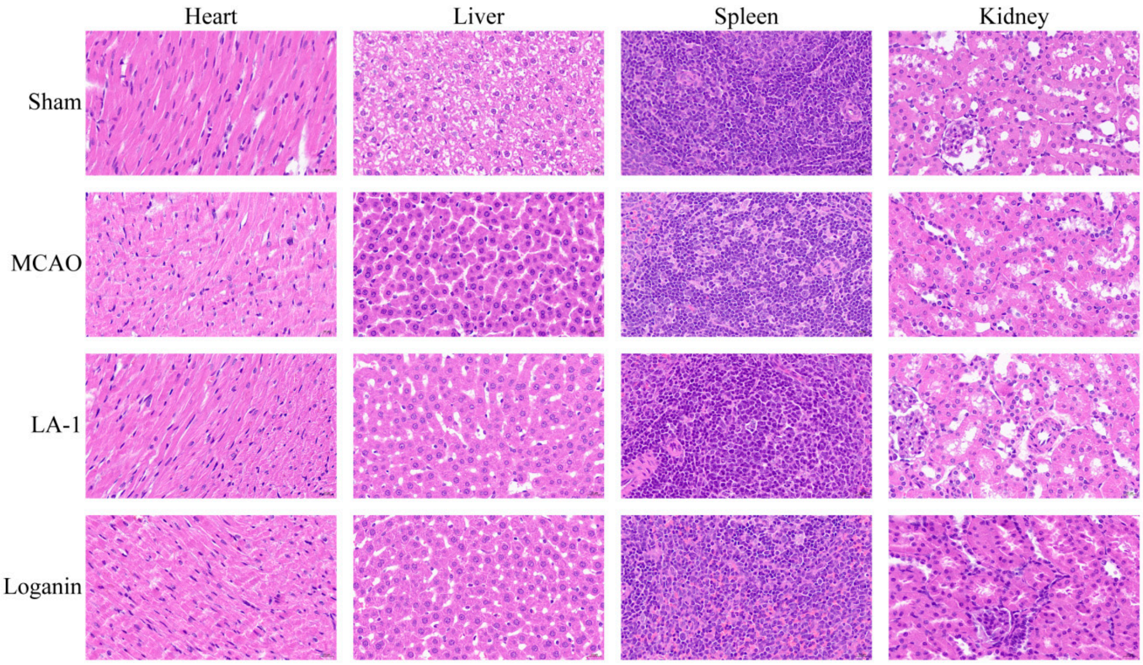


Figure S7 Assessment of the biosafety of drugs in vivo.
